# Operation in Cancer of the Uterus

**Published:** 1898-12-03

**Authors:** 


### OPERATION IN CANCER OF THE UTERUS.

IN his presidential address delivered before the Edinburgh Obstetrical Society, Dr. Halliday Croom touched upon many topics, and among them on the very serious and important question of the operative treatment of carcinoma of the uterus. The great improvements which have taken place during the past few years in the technique of the operation have made the removal of the uterus by way of the vagina a comparatively simple affair, and one not attended with much danger. Dr. Croom said that it was now 13 years since he did his first vaginal hysterectomy for cancer. That operation lasted two hours and a half; while the last one he did was performed in 18 minutes. Such was the advance in technique. But the question was, Did the operation, even when so shortened and simplified, tend much to prolong the life of the patient, or to ameliorate her sufferings? This Dr. Croom very much doubted, and for this reason, that the disease after hysterectomy proceeded rapidly to the peritoneum, where it produced for the patient greater sufferings and a death worse than if no operation had been done. True, he said, a vaginal hysterectomy stopped hæmorrhage and fœtid discharge, and relieved pain, but this was only a temporary improvement; and, although even a temporary alleviation of those symptoms would be an advantage if there were nothing on the other side, it was generally the case that with the recurrence of the disease the ultimate pain and suffering were far greater than if the disease had never been touched at all. Of 260 cases of uterine cancer which he had seen during 20 years only 15 fulfilled all the conditions requisite to justify hysterectomy with expectation of success. These were all operated on, and all made "absolutely uneventful recoveries"; but every one of them died within the year, and, in his opinion, with greater suffering than if they had been left alone. This is very disheartening, but if this is the present position of gynæcological knowledge it is best that the truth should be recognised. It is well known, however, that this view, as set out by Dr. Croom, is a gloomier one than is adopted by some other operators. No doubt much of the discrepancy between different observers on this subject may be due to the varying period at which the disease has been recognised by them, perhaps some to the fact that different observers demand different degrees of certitude before they pronounce the character of the disease in such cases. In the operative treatment of cancer of the uterus, as of other cancers, the thing of prime importance is the early recognition of the disease. One can see no reason why in really early cases removal of the uterus should not be at least as satisfactory as the removal of other organs so affected, and where the uterus is freely moveable, and signs of glandular implication are absent, we believe that the common practice is to urge hysterectomy. Under such circumstances it is a matter of grave moment to be told by a man of Dr. Halliday Croom's experience that in several of the cases mentioned by him the disease was still confined to the fundus, there being no involvement of the broad ligament, and that they were, therefore, typically desirable cases on which to operate, and yet that in all of them the disease recurred in the peritoneum, and the patient died in great distress within the year.

